# Technologies for Interoperable Internet of Medical Things Platforms to Manage Medical Emergencies in Home and Prehospital Care: Protocol for a Scoping Review

**DOI:** 10.2196/40243

**Published:** 2022-09-20

**Authors:** Mattias Seth, Hoor Jalo, Åsa Högstedt, Otto Medin, Ulrica Björner, Bengt Arne Sjöqvist, Stefan Candefjord

**Affiliations:** 1 Department of Electrical Engineering Chalmers University of Technology Gothenburg Sweden; 2 Prehospen – Centre for Prehospital Research Faculty of Caring Science, Work Life and Social Welfare University of Borås Borås Sweden; 3 InterSystems Corp Stockholm Sweden; 4 Äldre Samt Vård och Omsorgsförvaltningen Gothenburg Sweden

**Keywords:** interoperability, Internet of Medical Things, prehospital, home care, reference models, mapping, technologies, scoping review, falls, cardiovascular disease, stroke, medical emergency, home care, prehospital care

## Abstract

**Background:**

Population growth and aging have highlighted the need for more effective home and prehospital care. Interconnected medical devices and applications, which comprise an infrastructure referred to as the Internet of Medical Things (IoMT), have enabled remote patient monitoring and can be important tools to cope with these demographic changes. However, developing IoMT platforms requires profound knowledge of clinical needs and challenges related to interoperability and how these can be managed with suitable technologies.

**Objective:**

The purpose of this scoping review is to summarize the best practices and technologies to overcome interoperability concerns in IoMT platform development for medical emergencies in home and prehospital care.

**Methods:**

This scoping review will be conducted in accordance with Arksey and O’Malley’s 5-stage framework and adhere to the PRISMA-P (Preferred Reporting Items for Systematic Reviews and Meta-analyses Protocols) guidelines. Only peer-reviewed articles published in English will be considered. The databases/web search engines that will be used are IEEE Xplore, PubMed, Scopus, Google Scholar, National Center for Biotechnology Information, SAGE Journals, and ScienceDirect. The search process for relevant literature will be divided into 4 different steps. This will ensure that a suitable approach is followed in terms of search terms, limitations, and eligibility criteria. Relevant articles that meet the inclusion criteria will be screened in 2 stages: abstract and title screening and full-text screening. To reduce selection bias, the screening process will be performed by 2 reviewers.

**Results:**

The results of the preliminary search indicate that there is sufficient literature to form a good foundation for the scoping review. The search was performed in April 2022, and a total of 4579 articles were found. The main clinical focus is the prevention and management of falls, but other medical emergencies, such as heart disease and stroke, are also considered. Preliminary results show that little attention has been given to real-time IoMT platforms that can be deployed in real-world care settings. The final results are expected to be presented in a scoping review in 2023 and will be disseminated through scientific conference presentations, oral presentations, and publication in a peer-reviewed journal.

**Conclusions:**

This scoping review will provide insights and recommendations regarding how interoperable real-time IoMT platforms can be developed to handle medical emergencies in home and prehospital care. The findings of this research could be used by researchers, clinicians, and implementation teams to facilitate future development and interdisciplinary discussions.

**International Registered Report Identifier (IRRID):**

DERR1-10.2196/40243

## Introduction

### Background

Advancements in wireless technology, artificial intelligence, and sensor technology have enabled the use of remote patient monitoring as a method to prevent and detect medical emergencies [1]. This includes, for example, cardiovascular diseases [2], stroke [3], sepsis [4], and trauma, including falls, which together claim millions of lives each year [5-7]. These medical emergencies can often be attributed to population aging, as elderly individuals are more susceptible to disease and disability. Between 1990 and 2017, the 2 main causes of disease-specific deaths globally attributed to population aging were ischemic heart disease (3.2 million) and stroke (2.2 million) [8]. Other diseases that notably contribute to deaths among older adults (≥65 years) are heart failure (20%), dementia (13.6%), chronic lower respiratory disease (12.4%), and pneumonia (5.3%) [9]. Furthermore, degenerative diseases and arthritis gradually decrease individuals’ physical and mental capacities and have all been associated with a high incidence of life-threatening falls among elderly individuals [10,11]. As of 2021, falls are the second leading cause of all unintentional injury deaths worldwide [12].

Globally, medical emergencies impose a great economic burden. In 2015, the estimated medical costs attributable to fatal and nonfatal falls among elderly individuals (≥65 years) in the United States were approximately US $50 billion [13]. The estimated global cost of stroke is over US $891 billion, which is 1.12% of the global gross domestic product (GDP) [14]. Population growth and population aging further indicate that additional economic strain will be put on future health and social systems [15]. In 2020, Li et al [16] showed that the health care expenditure per capita in China of the age group ≥65 years was 7.25 times higher than the health care expenditure per capita of the age group ≤25 years. In the United States, people aged 55 and over account for more than half of total health spending [17]. In Sweden, the municipalities’ total cost of elderly care in 2020 was 135 billion SEK (approximately US $14 billion), an increase of more than 40% from the costs of 96 billion SEK (approximately US $10 billion, inflation considered) in 2010 [18].

Supporting the health and well-being of a growing population in the context of an aging population remains one of today’s most complex and critical global challenges [19]. More health care must be provided closer to patients’ homes to reduce health care costs and optimize health care processes [20]. This transition of health care motivates the need for technical solutions that can support its success. Several interconnected medical devices and applications, an infrastructure referred to as the Internet of Medical Things (IoMT), are suitable approaches for remote patient monitoring [21]. IoMT can increase patient safety, reduce health care costs, and streamline processes and workflows in home and prehospital care [1]. In the IoMT, devices communicate over the internet to achieve a common goal [22,23]. Furthermore, combining several devices, followed by adequate data fusion, can be advantageous in terms of system accuracy [24].

**Figure 1:**
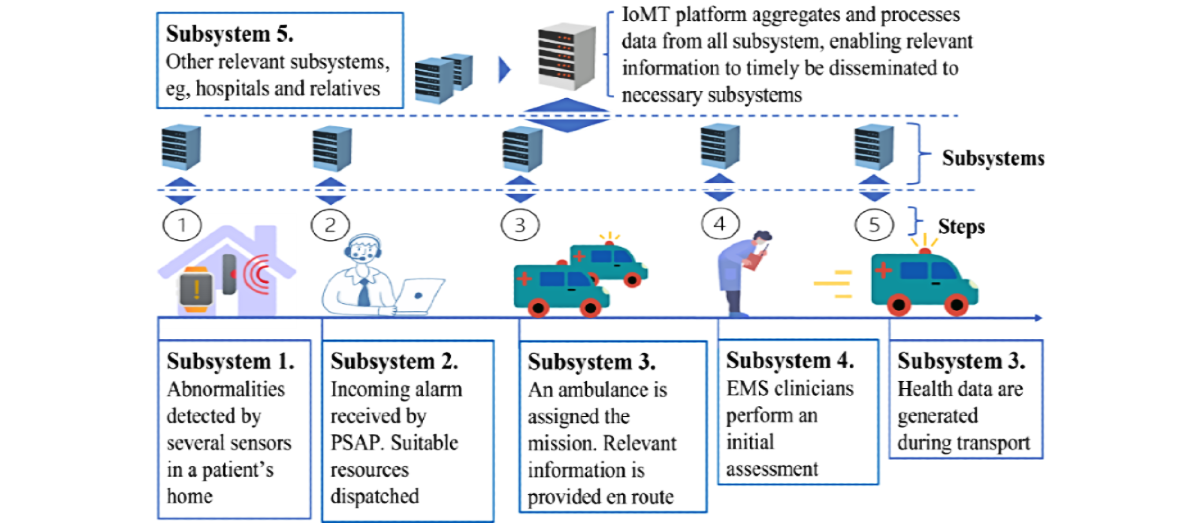
Example of events and data being transferred through several subsystems in home care and prehospital care settings. EMS: emergency medical services; IoMT: Internet of Medical Things; PSAP: public safety answering point.

Home care is the provision of health care in patients’ homes with the goal of complementing and replacing hospital care and improving quality of life [25] (Step 1 in Figure 1). Prehospital care refers to emergency medical services (EMS) provided to a trauma victim before they arrive at the hospital (Steps 2-5 in Figure 1). Together, home care and prehospital care include several steps: remote monitoring, health status assessment, resuscitation, and stabilizing measures [26]. Each step is associated with data generation and data processing. The sensors in Step 1 in Figure 1 can be deployed in a patient’s home, and in the case of detected abnormalities, an alarm can be sent to the public safety answering point (PSAP). For an adequate care process and patient safety, the information must rapidly and securely flow through each step in Figure 1.

Today's systems often lack the functionality necessary to manage all the steps depicted in Figure 1 [27]. Some studies have focused on different algorithms (eg, predicting the need for critical care [28], fall detection and fall prevention [29-31], certain sensor setups, pressure sensors [31], and radio frequency [32,33]) or certain levels of interoperability (semantic interoperability [34-37]). For systems to function effectively in both home and prehospital care, the whole scenario must be considered, and several interoperability challenges must be addressed. These challenges include (1) interoperability of the real-time data collection system, involving integration of devices and platforms from different vendors, allowing data fusion as a technique to increase system accuracy (Step 1, Figure 1) [24]; (2) interoperability in the data stored in disparate systems, such as medical devices, electronic health records (EHRs), emergency service centers, and emergency medical dispatch systems (subsystems, Figure 1); (3) definition of mechanisms for the dissemination of data to third-party applications; and (4) services for big data processing and knowledge extraction. According to Rubí and Gondim [27], prior studies have partially solved these challenges, although without considering the whole scenario. For information to flow through each step in Figure 1, all challenges 1-4 must be solved. In this scoping review, the focus is on interoperable IoMT platforms that address the interoperability challenges 1-4 and cover Steps 1-3 in Figure 1.

### The ASAP (Acute Support, Assessment, and Prioritizing) Project

The World Health Organization (WHO) has declared falls as a major public health problem [12]. Over 37 million severe falls are reported globally each year among older adults (≥65 years). Approximately 684,000 individuals die from falls each year, a number that is projected to increase due to population aging [12]. Hip fractures, traumatic brain injuries, and upper limb injuries are all examples of injuries following severe falls, and if appropriate treatment is not received in time, the injury can worsen [38]. Various postfall syndromes, such as confusion, immobilization, and depression, may place further constraints on daily activities several months after the fall [12]. According to the Swedish National Board of Health and Welfare, the costs for falls in Sweden amount to 17 billion SEK (approximately US $1.7 billion) [39].

**Figure 2:**
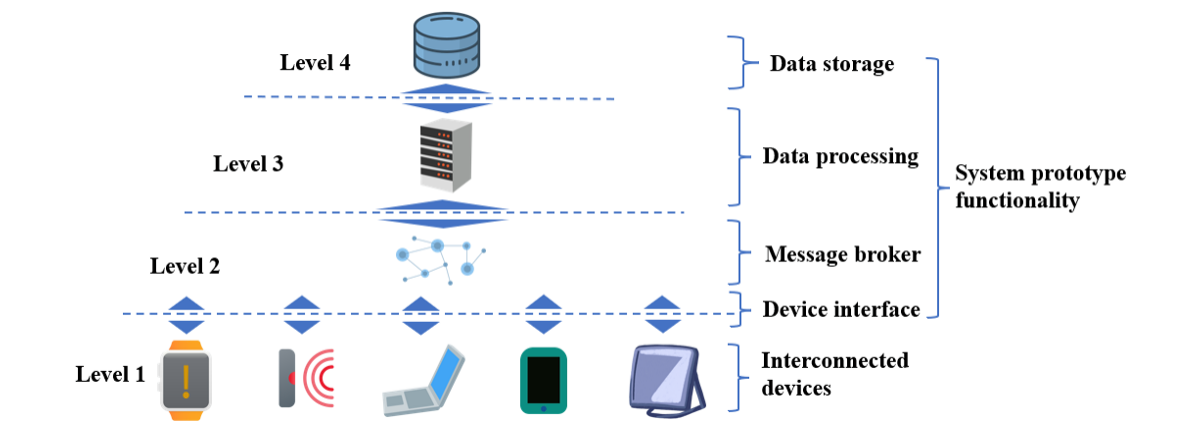
Simplified architecture visualizing the building blocks of the Internet of Medical Things (IoMT). In the ASAP (Acute Support, Assessment, and Prioritizing) project, the aim is to develop an interoperable system prototype that includes functions covered by Levels 2–4.

As a response to population aging and the need for more efficient home and prehospital care, an ongoing research project led by Chalmers University of Technology in Gothenburg, Sweden, aims to tackle these concerns in a project named ASAP (Acute Support, Assessment, and Prioritizing). Since falls account for 40% of all injury-related deaths among persons aged 85 years or older [10], the ASAP project’s initial focus is on falls. The aim of the ASAP project is to develop an interoperable system prototype (Figure 2) for home care and prehospital care, meaning that the system prototype will encompass functionalities necessary to manage Steps 1-3 in Figure 1, from the moment a person falls in their home until the paramedics arrive at the scene. Even though falls are the platforms’ initial focus, functionalities to manage additional medical emergencies such as congestive heart failure, arrhythmia, and stroke will be targeted in future system development processes.

### Interoperability Model

**Figure 3:**
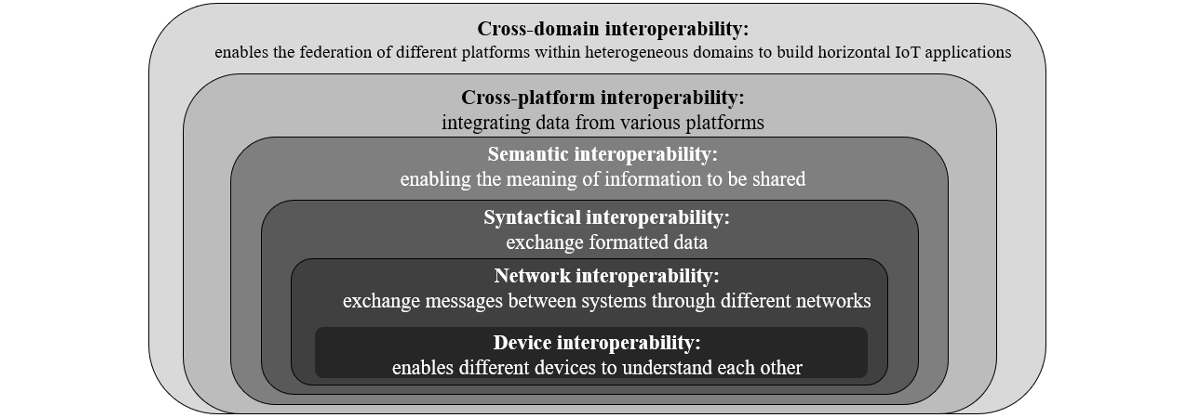
Interoperability model visualizing the different levels of interoperability. Each level is associated with different interoperability challenges.

Today, a great deal of health-related information is hidden in isolated data silos and incompatible systems, making it difficult to access and use this information [27]. However, medical emergencies require that information be exchanged rapidly and securely between systems [38]. For this interplay to function adequately, different devices and applications must be interoperable; they must access, exchange, and use information in a predictable and standardized manner [40-42]. Interoperability has recently received increased attention due to the need to uncover the full potential of big data and improve digital health. However, the precise meaning of the term interoperability is ambiguously defined [41,42]. Several definitions exist, and numerous attempts have been made to present the concept using different models [42]. In this scoping review, the term interoperability is conceptualized through a 6-level hierarchical structure (Figure 3) [42].

### Internet of Things (IoT) Architecture and Reference Models

**Figure 4:**
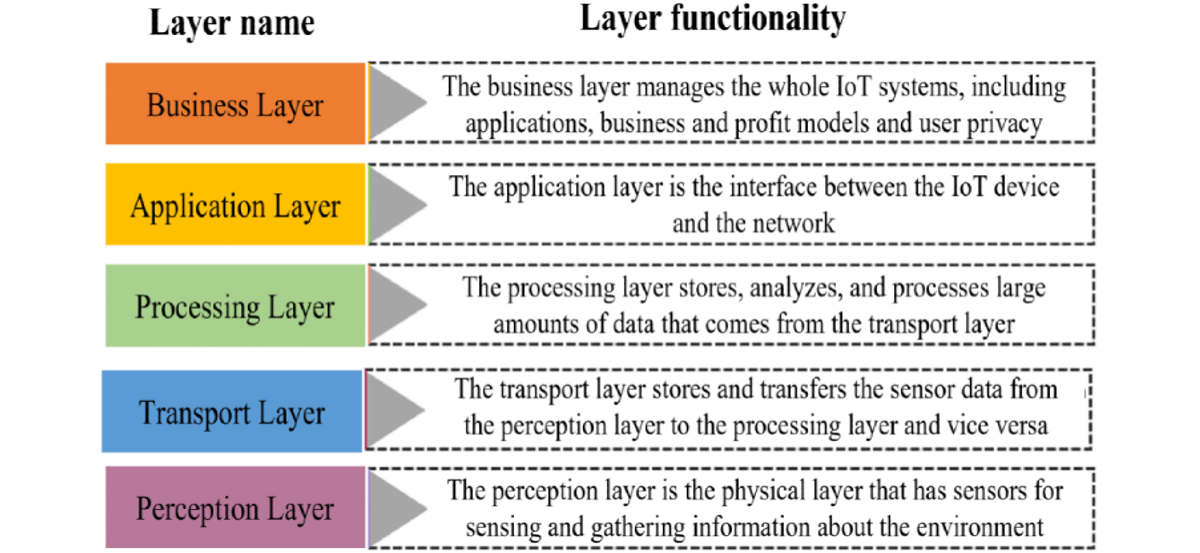
Different levels of an Internet of Medical Things (IoMT) reference model. Each layer is associated with different protocols and technologies, all of which help realize each layer’s specific functionality. The model helps to conceptualize IoMT building blocks.

Software-defined networking (SDN) [42-44] and computational infrastructures (such as fog computing [45,46]) have been identified as potential technologies needed to cope with latency and bandwidth problems due to congested networks [43,45]. These and other technologies have the potential to solve many of today’s interoperability challenges. Different protocols, a set of rules that allow machines and applications to exchange information [47], also play a key role in solving interoperability challenges. Together, different protocols form reference models (also called protocol stacks, Figure 4), which provide a structured way to discuss system components and system functions [23,47]. Knowledge of these models and technologies can facilitate the development of IoMT architectures [23] (Figure 2).

In this scoping review, technologies refer to approaches used to implement the IoMT building blocks in Figure 2. The IoMT reference model in Figure 4 further helps to conceptualize these building blocks. Technologies are limited to data formats and protocols (Level 1), middleware technology and application programming interfaces (APIs; Levels 2-4), computational infrastructures, data processing techniques (Level 3), data storage (Level 4) and standards, and network architectures (Levels 1-4). Hardware, project management processes, and regulatory compliance are not considered. The aim is to provide recommendations regarding suitable technologies that can be used to develop interoperable IoMT platforms and help to achieve the levels of interoperability presented in Figure 3.

### Previous IoMT Platform Development

Efforts from digital health global research communities have addressed concerns related to remote patient monitoring. Takatou and Shinomiya [48] developed an IoMT-based real-time fall detection prototype system for elderly individuals in 2020 using passive radio frequency identification (RFID) sensor tags. Rachakonda et al [49] presented Good-Eye in 2020, an IoMT-enabled device that can both detect and predict fall-related accidents using data fusion techniques. Good-Eye was able to predict falls with an accuracy of 95%. For validation of the Good-Eye system, 6 study participants and 144 different instances of sitting and falling were recorded with the use of depth cameras. Kommey et al [50] proposed a patient medical emergency alert system (PMEAS) that allows body temperature and heart rate to be collected and transmitted to the user’s phone via Bluetooth. The PMEAS accurately recorded the temperature of the user approximately 80% of the time [50]. Vandenberk et al [51] developed DHARMA, a component-based digital research platform for mobile remote monitoring studies. The DHARMA platform performed well in a real-time health care setting for the follow-up of pregnant women at risk of developing preeclampsia.

Although many solutions have promising results regarding the detection of abnormal values [49-53], research tends to focus on certain aspects of the problem concerning remote patient monitoring. For an IoMT platform to be integrated and employed in real-life settings, these platforms must be capable of managing multiple devices and their different characteristics (ie, different communication protocols and data formats) [54]. The lack of standards for data integration and deficient integration capabilities with external platforms and EHR systems impedes the usage of IoMT platforms in real life [40,54]. To date, little attention has been given to real-time systems that can be deployed in real-world care settings [27]. Therefore, more comprehensive studies that focus on effective and secure data transmission and management are needed.

### Related Work

Related studies have previously been conducted. Ait Abdelouahid et al [55] focused on a map between IoT communication protocols and an 8-level interoperability model. Garai and Adamkó [56] mapped a 3-level interoperability model against the 7-layer Open Systems Interconnection (OSI) model. Tayur and Suchithra [57] examined current standards, data formats, and protocols to overcome interoperability issues, focusing on the application layer. Leite et al [58] and Noura et al [42] examined technologies used to overcome interoperability concerns in the IoT. Sethi and Sarangi [23] presented a survey of the current technologies used in the IoT domain as of 2016. To our knowledge, there has been no previous study that combines interoperability challenges and suitable technologies with a focus on medical emergencies. This type of study is necessary to obtain an overview of how interoperable real-time IoMT platforms can be developed, which types of interoperability issues can arise, and how these can be managed with suitable technologies.

### Aim

This scoping review aims to summarize suitable technologies and best practices used during the development process of real-time interoperable IoMT platforms, with a focus on platforms that can handle medical emergencies, such as falls, congestive heart failure, and stroke, in home and prehospital care settings. The overall goal is to summarize and describe technologies used to overcome interoperability concerns. Furthermore, the aim is to provide recommendations regarding the technologies used to develop interoperable IoMT platforms, enabling clinicians and practitioners to understand relevant challenges and use appropriate techniques to tackle these concerns. Technical concepts will be described based on how they can be used in health care, enabling clinicians and nontechnical professionals to understand their application areas.

## Methods

### Overview

Arksey and O’Malley [59] describe 4 common reasons why it can be worthwhile to undertake a scoping review. The third reason is “to summarize and disseminate research findings” [59]. This description can best be applied to this scoping review. Hence, the aim is to summarize and disseminate research findings within the frontiers between clinical applications, interoperability, and IoMT platform development. Our scoping review protocol will be based on Arksey and O’Malley’s 5-stage methodological framework for scoping reviews [59]. In this model, Stage 1 consists of identifying the research question. Stage 2 involves identifying the relevant studies. Stage 3 comprises study selection. Stage 4 consists of charting relevant data from the studies. Stage 5 consists of collecting, summarizing, and reporting the results

If necessary, these stages will be further broken down into more manageable substeps to increase the transparency of the method. The electronic databases/web search engines used in the study are IEEE Xplore, PubMed, Scopus, Google Scholar, NCBI, SAGE Journals, and ScienceDirect, as these search engines have been previously used by related studies.

The scoping review protocol is reported in accordance with the reporting guidelines provided in the Preferred Reporting Items for Systematic Reviews and Meta-analyses Protocols (PRISMA-P) statement. The PRISMA-P checklist was developed for a systematic review protocol; therefore, not all items will be covered (see Multimedia Appendix 1).

### Stage 1: Identification of the Research Question

**Figure 5:**
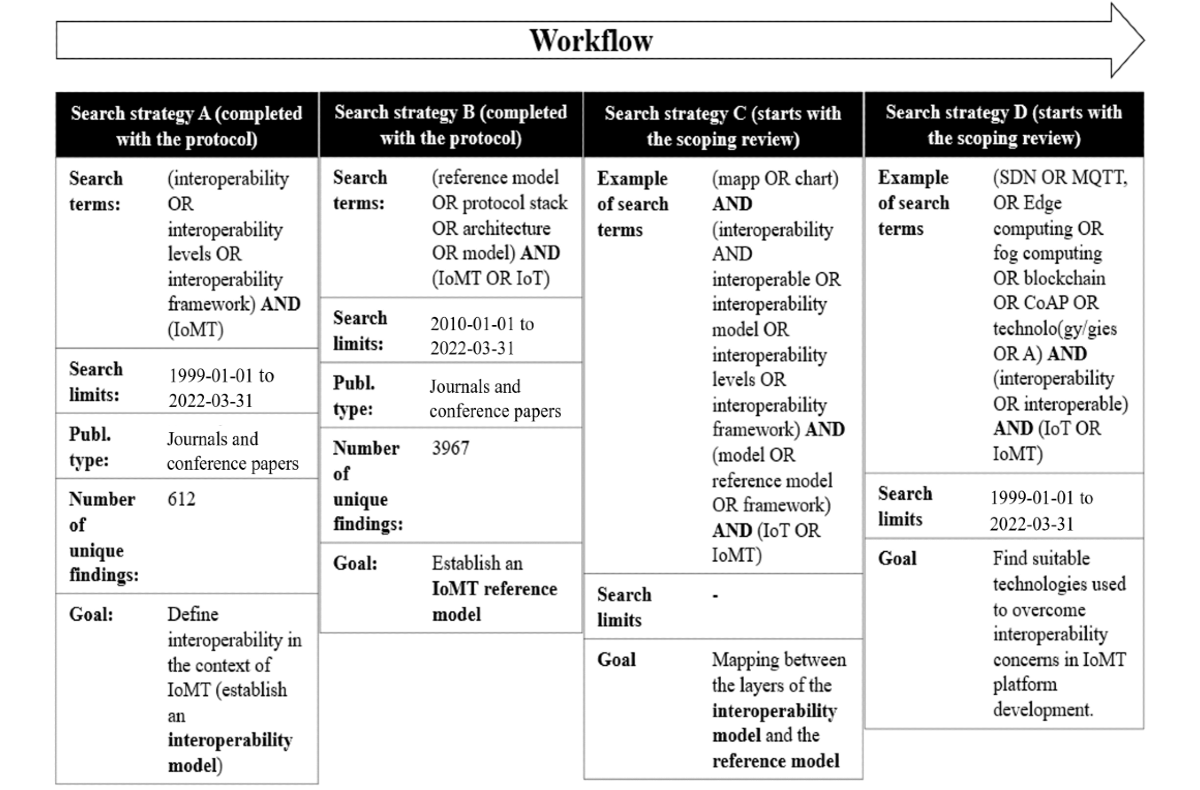
The different search strategies. IoMT: Internet of Medical Things; IoT: Internet of Things; MQTT: Message Queuing Telemetry Transport; SDN: software-defined networking.

A preliminary search of the literature was conducted. The aim was to outline important keywords to refine the search terms used in the review and to develop relevant research questions. Studies were screened by title and abstract to determine suitability for inclusion. Several abbreviations, terms, and acronyms that were found in the literature and deemed to be relevant were noted and tabulated (Textbox 1). To decide whether a term, abbreviation, or acronym should be tabulated, it had to fulfill 2 criteria. First, it had to be considered a keyword that could help the reviewers search for literature that could be used to identify relevant research questions. Second, it had to be a new keyword. These new keywords were, however, combined with other familiar search terms using the Boolean operators AND and OR (Figure 5). Stage 1 was completed with this scoping protocol.

In addition, a snowball approach described by Wohlin [60] was used to further screen for new articles. Snowballing refers to using the reference list of a paper (backward snowballing) or the citations to the paper (forward snowballing) to identify additional papers [60]. Both backward and forward snowballing were used in Stage 1. The identified articles were continuously saved to the reference manager Mendeley (version 2.70; Elsevier), and duplicates were removed.

**Examples of abbreviations, terms, and acronyms relevant for Internet of Medical Things (IoMT) platform development.**

**Standards**

- FHIR (fast health care interoperability resources)
- oneM2M
- openEHR
- SenML (Sensor Markup Language)

**Network/software architectures**

- Multitenancy
- SDN (software-defined networking)
- SOA (service-oriented architecture)

**Internet of Medical Things (IoMT) platform/software frameworks**

- Aneka
- FogBus
- GiraffePlus
- GoodEye
- HealthGo
- HPCaaS (high-performance computing as a service)

**Protocols**

- AMQP (Advanced Message Queueing Protocol)
- CoAP (Constrained Application Protocol)
- IPv6 (Internet Protocol version 6)
- MAC (media access control)
- MQTT (Message Queuing Telemetry Transport)
- RDP (Remote Desktop Protocol)
- SCAIP (Social Care Alarm Internet Protocol)
- Websockets
- XMPP (Extensible Messaging and Presence Protocol)

**Computational infrastructures/systems**

- Apache Flink Apache Kafka
- Apache Storm
- Fog computing
- Multiaccess edge computing
- Edge computing

**Data management**

- Blockchain
- Stream reasoning

The following research questions were identified:

1. What are the current challenges of developing a real-time IoMT platform for managing medical emergencies such as falls?
2. What is interoperability, and how can it be defined in the context of the IoMT?
3. What types of models are used to visualize the different layers of interoperability? When talking about medical devices in an IoMT setting, which model is preferable and why?
4. Which reference model with corresponding protocols can best describe and define the structure of key aspects of the information being managed in a real-time IoMT system? How is the model being used today?
5. Have any studies examined which current technologies are associated with the layers in the reference models identified in research question 3, and how these are being used to fulfill the set of rules defined by each layer? If so, what are the results?
6. How can interoperability solutions be mapped to the layers in the interoperability model?
7. What recommendations regarding technologies can be given to clinicians and practitioners who want to develop interoperable IoMT platforms for home and prehospital care settings?

### Stage 2: Identification of Relevant Studies

To answer the research questions outlined in Stage 1, a comprehensive search strategy will be conducted. Since the scoping review encompasses a broad spectrum of research questions, the search process will be divided into 4 search strategies with separate search terms, search limits, and goals (Figure 5). Strategies A-D will be carried out in chronological order, starting with search strategy A. Search strategies A and B were completed with this protocol.

**Figure 6:**
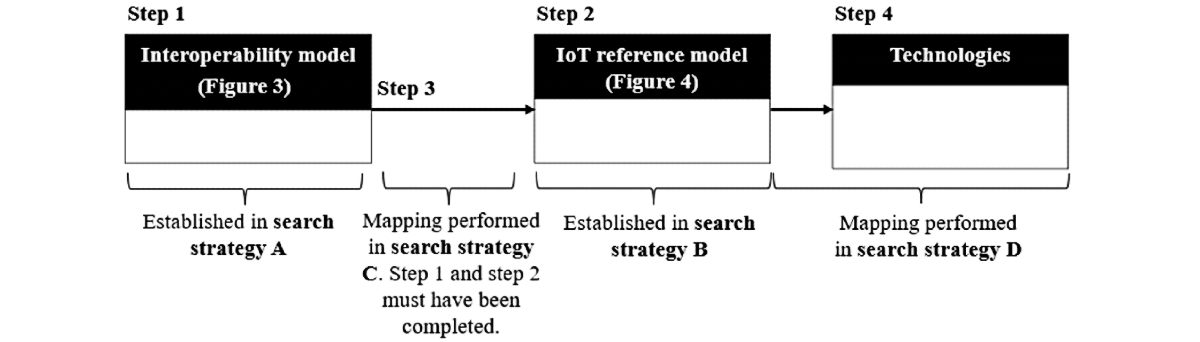
Reasoning behind the chronological workflow. The workflow is carried out in the following order: Step 1, Step 2, Step 3, and Step 4. IoT: Internet of Things.

The chronological workflow structures the work into more manageable pieces and ensures that any prior knowledge deemed necessary for each search strategy has been acquired in the previous step. Figure 6 shows the reasoning behind the chronological workflow and provides an overview of how the search strategies relate to each other.

Backward and forward snowballing was used in Steps A and B and will further be used in Steps C and D. The final inclusion of a paper will be based on the eligibility criteria in Textbox 2. These criteria will be applied in all steps.

**Inclusion and exclusion criteria. IoMT: Internet of Medical Things; IoT: Internet of Things.**

**Inclusion criteria**

- Published peer-reviewed journals and conference papers
- Written in the English language
- Published during the time period defined in the protocol
- Studies describing or reporting the development or design of IoMT systems with a focus on the technology
- Studies reporting challenges and barriers of integrating IoMT platforms into prehospital care or home care settings with a focus on the technology
- Studies describing different relevant technologies used for IoMT platform development

**Exclusion criteria**

- Full-text articles that could not be obtained and/or are not written in English
- Conference abstracts, book reviews, commentaries, and editorial articles
- Studies focusing on hardware, project management processes, or regulatory compliance
- Studies reporting on the design or development of IoT applications with no focus on health data (eg, Industry 4.0, including the automotive industry, food industry, manufacturing industry, etc).

#### Search Strategy A: Defining Interoperability

Search strategy A covered the concept of interoperability. It was completed with this protocol and resulted in the interoperability model (Figure 3). It laid the foundation regarding what interoperability concerns and helped establish an interoperability model that we can proceed from (Figure 3). Since the term interoperability is hard to define and numerous conceptual frameworks exist, the aim was to find a model best suited for the purpose of this scoping review (ie, reviewing software technologies associated with interoperability concerns in IoMT settings). Hence, the aim of strategy A was to cover articles published between January 1, 1999, and March 31, 2022, since the term “Internet of Things” appears to have been first coined in 1999 by Kevin Ashton [61], whereas the term “interoperability” appears to have been around since 1970 [62].

#### Search Strategy B: IoT Reference Models

Search strategy B covered reference models. It was completed with this protocol and resulted in the reference model (Figure 4). The focus was on models used to describe the interface between different components in an IoT setting, since knowledge of the architectural structure of reference models is necessary in software development [23]. Due to the rapid increase in the number of interconnected devices in recent years [63] and to limit the search results, search strategy B was limited to studies published between January 1, 1999, and March 31, 2022.

#### Search Strategy C: Mapping Between the Interoperability Model and the IoT Reference Model

**Figure 7:**
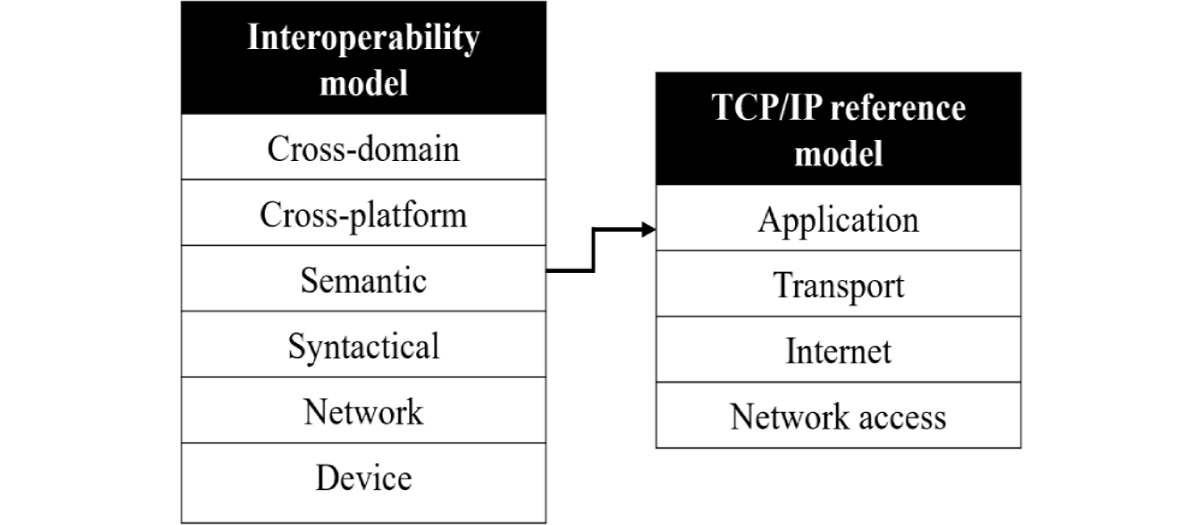
Example of mapping between the layers in an interoperability model and the layers in a Transmission Control Protocol/Internet Protocol (TCP/IP) model. In this case, challenges related to semantic interoperability can arise at the application layer of the TCP/IP model.

**Figure 8:**
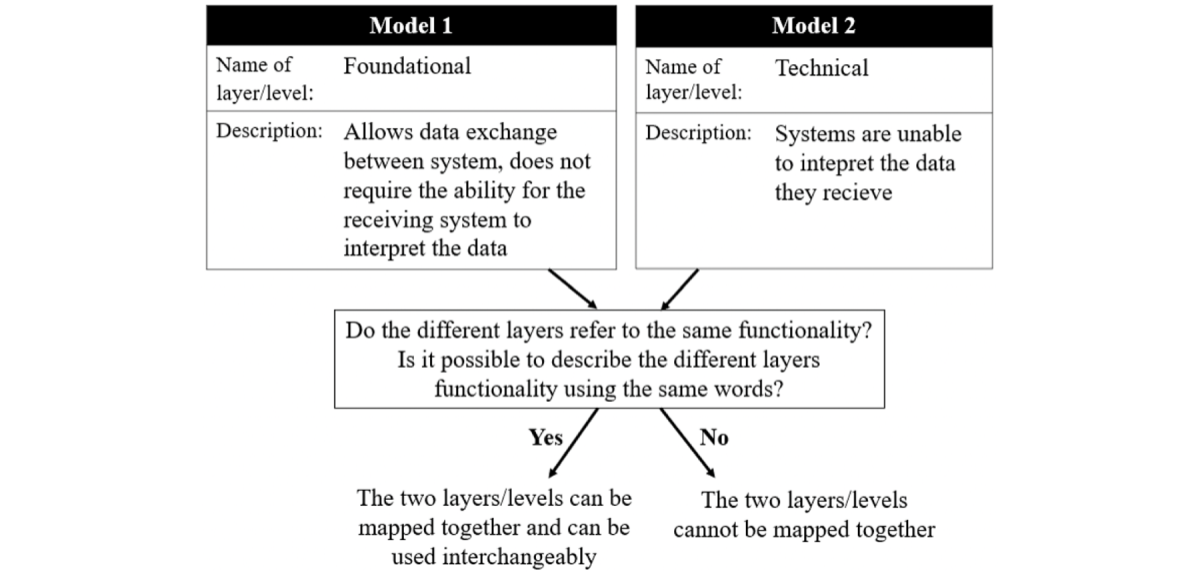
Schematic overview of the mapping process. In this case, the 2 layers can be mapped together.

In search strategy C, we will proceed from Figures 3 and 4. Strategy C will start with the scoping review. The goal is to map the levels in the interoperability model (Figure 3) to the corresponding levels in the reference model (Figure 4). Similar mappings as suggested in the literature will be examined and used as a reference [61]. Each mapping will be performed according to the layer(s) in the reference model in which different interoperability challenges appear (Figure 7). This will be followed by an explanation of the motivation regarding the terms on which the mapping has been performed. Two separate models can name their layers differently but describe the same concept or model functionality. Therefore, the mapping process will be systematically conducted following the process described in Figure 8.

#### Search Strategy D: Technologies

Strategy D will cover technologies used to overcome interoperability challenges during the development process of real-time interoperable IoMT platforms. In this step, we will proceed from Figure 3. Technologies will be tabulated and followed by a descriptive overview regarding how that technology can be applied in the context of IoMT platform development to handle medical emergencies (Figure 7). Search terms specified in Figure 5 will be complemented with terms from Textbox 1. Articles describing the development process of IoT platforms [64] will be of particular interest since these often cover several aspects regarding the development process and thus act as a source of information from which new technologies and search terms can be obtained.

The majority of standard IoT protocols were developed in the late 1990s or during the 21st century. For example, the Constrained Application Protocol (CoAP) was published as a full Internet Engineering Task Force (IETF) internet standard in 2014, the Advanced Message Queuing Protocol (AMQP) was created in 2003, and Message Queuing Telemetry Transport (MQTT) was created in 1999 [65]. Therefore, the search strategy will be limited to articles published between January 1. 1999, and December 31, 2022. This limitation is further motivated by the fact that new emerging concepts such as SDN (2008) [43], fog computing (2012) [66], and blockchains (2008) [67] have been introduced over the past 2 decades.

### Stage 3: Study Selection

Studies will be selected based on 2 screening processes. To facilitate the screening processes, several inclusion and exclusion criteria were developed (Textbox 2). Inclusion criteria will be adapted in both screening processes.

#### Title and Abstract Screening

The first screening process will include an evaluation and an assessment of the relevance of the articles’ titles and abstracts. Rayyan (Rayyan Systems Inc) will be used by 2 reviewers in the group (authors MS, HJ) to reduce any biases. Rayyan is a free mobile and web tool designed to help researchers work on knowledge synthesis projects, including scoping reviews [68]. The 2 reviewers will perform the screening process independently of each other. In case of any disagreements, a third member of the research team will vote regarding whether the article should be included. Inclusion or exclusion will then be based on the majority decision. All 3 reviewers will use the same inclusion and exclusion criteria when deciding whether an article should be included. Decisions will be based on majority vote.

#### Full-Text Screening

The articles that pass the title and abstract screening will undergo a second screening stage. This stage involves a full-text review conducted in the same way as the first screening process.

### Stage 4: Charting the Data

In this stage, only articles that pass title and abstract screening and full-text screening will be summarized. Information relevant for extraction will include general findings that are shared among the articles that are to be summarized. These findings include author(s), year of publication, country of origin, purpose/aim of the study, methodology, type of study, and outcomes. In addition to these general findings, more specific information that will help answer each research question will be summarized, including the following: (1) What technologies are used to develop real-time interoperable IoT/IoMT platforms? (2) How are the technologies used? (3) For what purposes are the technologies used? This will include information about the models used, technological approaches and their pros and cons, research context, challenges and barriers, conclusions, and future work.

To map the findings regarding technologies to corresponding layers in the reference model, the extracted data will be categorized using the web application Dedoose (SocioCultural Research Consultants), a qualitative data analysis application [69]. The focus will be on qualitative data. Quantitative evaluations or measurements of system performance will not be considered. The coding scheme will be tested by 2 separate members (authors MS, HJ) of the team to ensure that it is a suitable and applicable scheme.

### Stage 5: Collating, Summarizing, and Reporting the Results

In this stage, findings from the reviewed literature will be summarized and presented. The summary will include both a descriptive summary and a thematic analysis. Qualitative analysis techniques will be used to complete the thematic analysis. Models established in Stages 1 and 2 will be visualized through images and described in running text. The mapping conducted in search strategy C will be visualized through models and tables. Findings (technologies) from search strategy D will be tabulated together with a definition and a description in running text. Findings from search strategy D will also form the basis for recommendations on suitable technologies that can be used during the development of interoperable IoMT platforms.

The result will provide an overview of how and why different interoperability concerns appear at different stages during the software development process and how these can be managed through the usage of suitable technologies. If the same technology is examined and recommended in multiple studies, the number of occurrences will be summarized and reported. Although the focus is on real-time IoMT platforms, the result will hopefully be valuable to a broad audience working with IoMT applications. Because an assessment of study quality is not routinely used in scoping reviews [69,70], this kind of assessment will not be addressed in this scoping review.

### Ethical Considerations

The scoping review will build upon previously published papers where data from potential trials have already been ethically approved before commencement, so no additional ethical approval is necessary.

## Results

A preliminary search for potentially relevant articles was performed in April 2022 using the electronic databases IEEE Xplore, PubMed, Scopus, Google Scholar, National Center for Biotechnology Information, SAGE Journals, and ScienceDirect. A total of 4579 articles were found. The data extraction and analysis will be completed in early 2023. The qualitative and thematic analyses will be complemented by descriptive statistics and narrative form. We expect the results from this scoping review to be disseminated in a scientific peer-reviewed journal in 2023. The results will be disseminated through scientific conference presentations, oral presentations, and publication in a peer-reviewed journal.

## Discussion

### Expected Findings

In this scoping review protocol, we define interoperability in the context of IoMT and choose a 6-level model to conceptualize different interoperability issues that can arise during IoMT platform development. Additionally, we define a 5-level IoMT reference model to conceptualize building blocks of IoMT platforms. These definitions and building blocks will be the basis for our review, and data will be mapped to this structure.

From a clinical perspective, this scoping review will provide information necessary for building interoperable IoMT platforms for managing medical emergencies in home and prehospital care settings. To date, several reviews have been published regarding interoperability and IoMT platform development. However, to our knowledge, this will be the first review that combines interoperability and technologies with a focus on medical emergencies in prehospital and home care. The strengths of this study lie in the combination of an interoperability model and an IoMT reference model. By mapping interoperability issues to the layers in the IoMT model, we hope it will become clear how, where, and why different interoperability issues appear. Furthermore, this study will include suitable technologies to overcome these concerns, which will facilitate readers to familiarize themselves with important tools needed to realize interoperable IoMT platforms.

Since we find collaboration between clinicians and engineers important in the context of IoMT development, our goal is to explain concepts in a simple way and continually point out application areas and how the technologies can be used to realize an IoMT platform. Another strength with the study is that readers with a nontechnical background should be able to comprehend the content and become acquainted with important concepts. The scoping review will be a facilitator for future interdisciplinary discussions.

### Limitations

One limitation of this study is that because its focus is on technologies in IoMT settings, we have intentionally omitted articles covering technologies in other IoT settings. For example, IoT technologies predominated in automotive industry or Industry 4.0 will not be reviewed, even though they could potentially add value to the IoMT development process. Another limitation is that articles were collected from a limited set of literature resources from a specific time period and only those published in English. A limited search strategy can increase the risk of selection, retrieval, and publication bias. To reduce selection bias, however, the screening process will be performed by 2 reviewers.

### Conclusions

This scoping review has the potential to influence future directions and may impact future IoMT platform developing processes. The results will elucidate important tools and concepts and enable clinicians and technicians to work closely in future development processes.

## Appendix

Multimedia Appendix 1 PRISMA-P (Preferred Reporting Items for Systematic review and Meta-Analysis Protocols) 2015 Checklist.

## Abbreviations

AMQP: Advanced Message Queuing Protocol
APIs: application programming interfaces
ASAP: Acute Support, Assessment, and Prioritizing
CoAP: Constrained Application Protocol
EHR: electronic health record
EMS: emergency medical services
GDP: gross domestic product
IETF: Internet Engineering Task Force
IoMT: Internet of Medical Things
IoT: Internet of Things
MQTT: Message Queuing Telemetry Transport
OSI: Open Systems Interconnection
PMEAS: patient medical emergency alert system
PRISMA-P: Preferred Reporting Items for Systematic Reviews and Meta-analyses Protocols
PSAP: public safety answering point
RFID: radio frequency identification
SDN: software-defined networking
WHO: World Health Organization
